# Modeling Lactic Fermentation of Gowé Using *Lactobacillus* Starter Culture

**DOI:** 10.3390/microorganisms4040044

**Published:** 2016-12-01

**Authors:** Bettencourt de J. C. Munanga, Gérard Loiseau, Joël Grabulos, Christian Mestres

**Affiliations:** 1 Instituto Superior de Tecnologia Agro-Alimentar de Malanje—ISTAM, EN. 230, Cangambo, Malanje, Angola; kudymwena@yahoo.com.br; 2 Montpellier SupAgro, UMR Qualisud, Montpellier 34398, France; 3 CIRAD, UMR Qualisud, Montpellier 34398, France; joel.grabulos@cirad.fr (J.G.); christian.mestres@cirad.fr (C.M.)

**Keywords:** lactic acid bacteria, fermented food, cereal based product, growth, mathematical model

## Abstract

A global model of the lactic fermentation step of gowé was developed by assembling blocks hosting models for bacterial growth, lactic acid production, and the drop of pH during fermentation. Commercial strains of *Lactobacillus brevis* and of *Lactobacillus plantarum* were used; their growth was modeled using Rosso’s primary model and the gamma concept as a secondary model. The optimum values of pH and temperature were 8.3 ± 0.3, 44.6 ± 1.2 °C and 8.3 ± 0.3, 3.2 ± 37.1 °C with μ_max_ values of 1.8 ± 0.2 and 1.4 ± 0.1 for *L. brevis* and *L. plantarum* respectively. The minimum inhibitory concentration of undissociated lactic acid was 23.7 mM and 35.6 mM for *L. brevis* and *L. plantarum*, respectively. The yield of lactic acid was five times higher for *L. plantarum* than for *L. brevis*, with a yield of glucose conversion to lactic acid close to 2.0 for the former and 0.8 for the latter. A model was developed to predict the pH drop during gowé fermentation. The global model was partially validated during manufacturing of gowé. The global model could be a tool to aid in the choice of suitable starters and to determine the conditions for the use of the starter.

## 1. Introduction

Fermentation is one of the oldest methods of preserving food and involves microorganisms whose metabolic activity and growth determine both organoleptic properties and shelf-life [1]. Most fermented foods consumed worldwide are produced by lactic fermentation from different raw materials such as milk, meat, fish, vegetables, cereals, roots and tubers, with a prominent place for fermented starchy foods: cassava, maize, millet, rice and sorghum, which are an important part of the diet in developing countries [2,3].

In the past, the need to produce fermented foods in large quantities increased in parallel with the advancement of knowledge about the identity of microflora responsible for lactic fermentation. This led to the selection of microbial starters that allowed industrial-scale production of fermented food products of reliable and predictable quality from milk, cereals, and meat. Many of the processes used to prepare traditional fermented foods in developing countries are poorly understood and controlled [4], which limits the ability to produce food of controlled quality at an industrial scale, especially due to the lack of suitable microbial starters [5]. Whatever the context, improving the quality of fermented foods remains an important objective that can be achieved in the case of lactic acid fermentations by using starter cultures that lead to rapid acidification of the raw material used [6]. According to [7], modeling lactic fermentation must include the growth of microbial strains and their functional properties. Modeling can be a valuable tool to help select appropriate strains, design the right equipment, determine the values of the control parameters to ensure greater food security and the quality of end products, and reduce financial losses. For example, modelling the simultaneous effects of fermentation temperature, total solids level of milk and total inoculum level helped to optimize the rheological properties and acidification kinetics of milk fermentation with starter bacteria [8]. In the same way, predictive microbiology helped for the selection of suitable starters for table olive fermentation [9]. Historically, the first models of predictive microbiology were developed to predict the growth of pathogenic bacteria [10]. These models are very useful to describe and simulate the growth of a microbe in environmental conditions. The growth of a population can be modeled using mechanistic or empirical approaches but most models are not entirely one or the other [11]. Empirical models aim for the best fit to the observed data without explaining the phenomena causing the observed response. An example of a widely used empirical model is described in [12]. Mechanistic or semi-mechanistic models are based on knowledge of the biological processes involved, such as the model described in [13]. Predictive microbiology modeling uses primary and secondary models. Primary models, like in the useful logistic growth model proposed by [14], describe changes in the microbial population over time using various parameters such as the initial number of cells, the final number of cells and the maximum growth rate. Secondary models predict changes in the bacterial growth rate as a function of environmental factors such as temperature, pH, water activity (a_w_) oxygen tension, etc. In empirical models, the effects of the environmental factors are described simultaneously through a polynomial function or response surface models [15,16]. In the gamma concept, introduced by Zwietering et al. [17] and the cardinal model proposed by Le Marc et al. [18]), each environmental factor acts independently through empirical models, for example, polynomial functions, and their combined effect is multiplicative. In order to better describe and simulate the fermentation process, global models were tentatively built by including equations describing the consumption of substrates, the production of metabolites, changes in the physical and chemical characteristics of the raw material. However, most authors pointed to the difficulty of coping with the increasing complexity of the models and the need for multidisciplinary approach to build complex models that account for the main changes that occur during the fermentation process, while offering user friendly and easily adaptable models [7,19,20].

This paper reports on the first studies in a global project that aims to provide a global but simple model for optimizing batch fermentations such as the fermentation of gowé, a traditional fermented Beninese beverage made from malted and non-malted sorghum flour that is produced by spontaneous fermentation involving mixed cultures of lactic acid bacteria (LAB) and yeasts [21,22,23]. The predominant LAB in gowé are *Lactobacillus fermentum*, *Wissella confusa*, *Lactococcus mucosae*, *Pediococcus acidilactici*, *Pediococcus pentosaceus* and *Wissella kimchii* [24] and the predominant yeasts are *Kluyveromyces marxianus*, *Pichia anomala*, *Candida krusei*, *Candida tropicalis* and *Clavispora lusitaniae* [25]. The quality of gowé recognized by consumers is complex: it should have a light and smooth texture, sweet taste, the aroma of a fermented product but low acidity [23,24,25,26]. During the process of preparing gowé, amylolysis of starch, which gives the product its lightly sugary taste and its light texture, occurs during the fermentation step, but is rapidly inhibited by acidification caused by the growth of the lactic acid bacteria [27,28]. Controlling fermentation to satisfy consumer demand for quality of gowé is thus very complex, and a modeling approach would be very useful to identify the optimum conditions that meet consumer demand.

**Figure 1:**
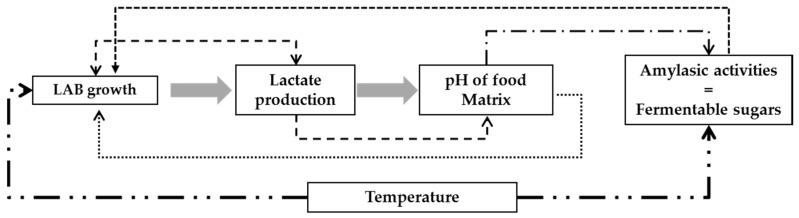
Schematic representation of the global fermentation model of gowé. Each block hosts the model of one process, and interactions are represented by the arrows with dotted lines. LAB: lactic acid bacteria.

The model is designed as an assembly of blocks as proposed by [29]. Each block hosts mathematical equations that model the reactions or processes linked to the fermentation process. In the case of the lactic fermentation of gowé, the main blocks are (Figure 1) the kinetic of lactic acid bacteria growth, the production of lactic acid, the calculation of the pH value, the activity of amylase, and the release of free sugars from starch due to the action of amylase. Undissociated lactic acid and the drop in the pH have a retro-action on both bacterial growth and amylase activity [27]. The temperature influences the whole set of biological reactions.

In this first study, we focused on the first three blocks linked to LAB growth; starch amylolysis and yeast growth models will be presented in following papers. Growth models of *Lactobacillus brevis* (CNCM I-2002) and *Lactobacillus plantarum* (CNCM I-3069) in De Man Rogosa and Sharpe (MRS) broth were built using Rosso’s primary model [30] and the gamma concept for the impact of pH, lactic acid content and temperature. A semi-mechanistic model predicting a drop in the pH of sorghum flour and malt due to acid production was developed and, the predictability of the model was evaluated during gowé fermentation using raw materials decontaminated by irradiation.

## 2. Materials and Methods

### 2.1. Raw Material

Grains of red sorghum (*Sorghum bicolor*, *(Linnaeus) Moench*), traditionally used for preparing gowé, were purchased at the local market in Cotonou (Benin). Raw sorghum flour was prepared by directly grinding the grains in a Laboratory Mill 3100 (Perten Instruments, Hagersten, Sweden) equipped with a 0.5 mm sieve. Malting was performed at the laboratory as detailed in [27], and the malted grains were ground in the same way as raw grains.

Malted and non-malted sorghum flours were treated by gamma irradiation with 2 kGy (Ionisos Company, Danieux, France). After this treatment, the residual yeast population was 1.3 × 10^4^ colony forming unit (CFU)·g^−1^ and the residual lactic acid bacteria population was below the threshold of detection (30 CFU·g^−1^)

### 2.2. Microbial Strains

*L. brevis* (CNCM I-2002) and *L. plantarum* (CNCMI-3069) were provided by Lesaffre, (Marcq-en-Baroeul, France) as dry active bacteria stored at 4 °C. For all experiments, dry active bacteria were inoculated directly.

### 2.3. Microbiological Methods

#### 2.3.1. Batch Cultures

Six-hundred-milliliter glass double wall fermenters with a useful volume of 300 mL without aeration were used. The temperature of the outer jacket was controlled with a thermostatic water bath. Mild but constant homogenization was achieved with a magnetic stirrer. The pH of the media was monitored and automatically adjusted with sterile 1 M sodium hydroxide. Bacterial growth in MRS broth pH 6.4 (Biokar-diagnostics, Beauvais, France) was monitored using an in-line near infrared turbidity sensor (Optek FC20-ASD19-N, Elscolab, Arcueil, France). Cell count was expressed as CFU·mL^−1^ according to a calibration curve that was pre-established for each strain.

For pilot manufacturing of gowé, the medium was a mixture of 25 g of malted sorghum flour and 75 g of non-malted sorghum flour dispersed in deionized water. One part (15 g) of the non-malted flour was pre-cooked with 75 mL of deionized water at 65 °C for 10 min. When the temperature dropped to 30 °C, the rest of the malt flour, non-malted flour, and 125 mL of demineralized water was added. In this case, the bacterial load was monitored using the plate counting method.

For the determination of the minimum inhibitory concentration (MIC) of undissociated lactic acid, tubes containing MRS broth buffered at pH 5 (with 1 M phosphate buffer) and lactic acid concentrations ranging between 0 and 50 mM were inoculated with *L. brevis* or *L. plantarum* at a concentration of 10^6^ CFU·mL^−1^. The tubes were incubated at 37 °C, and growth was visually evaluated after five days. The final pH was measured, and ranged between 4.7 and 4.8.

#### 2.3.2. Plate Counting Methods

Tenfold serial dilution of homogenized samples (0.1 mL) was prepared in sterile 9% NaCl water and plated on the surface of De Man Rogosa and Sharpe agar plates (MRS-agar, pH 5.7, Biokar-diagnostics, Beauvais, France) for LAB and on Sabouraud chloramphenicol agar medium (Biokar-diagnostics, Beauvais, France) for yeasts. The MRS plates were incubated at 37 °C for 48 h. Sabouraud plates were incubated at 30 °C for 48 h.

### 2.4. Chemical Analyses

Lactic acid and glucose contents were measured by HPLC with separation on an Aminex HPX-87H column (Biorad, Hemel Hempstead, UK. and detection with both refractive-index and ultraviolet (UV) (210 nm) detectors. Elution was performed at 30 °C with 5 mM sulfuric acid at a flow rate of 0.6 mL·min^−1^. Samples were centrifuged at 7200 rpm for 5 min and filtered through 0.45 μm pore size filter before injection.

### 2.5. Mathematical Modeling

#### 2.5.1. Primary and Secondary Models

The logistic growth model with delay [14] was chosen to describe microbial growth according to Equation (1):

(1) $$\{\begin{array}{c} \frac{dNt}{dt}=0\;ift\leq \lambda \\ \frac{dNt}{dt}=\mu_{\max}Nt\left(1-\frac{Nt}{N_{max}}\right)ift>\lambda \end{array}$$

where *Nt* and *N_max_* (CFU/mL) are the values of microbial population at time *t* and at the end of the growth curve, respectively, µ_max_ the maximal growth rate (h^−1^) and *λ* the lag time (h).

The gamma concept model [17] was used as secondary model to describe the impact of temperature, pH and the undissociated lactic acid concentration ([*AH*]) on the maximum growth rate (μ_max_) according to Equation(2).

(2) $$\mu_{\max}=\mu_{\mathrm{opt}}*\gamma \left(T\right)*\gamma \left(\mathrm{pH}\right)*\gamma \left(\left[AH\right]\right)$$

with *γ* values ranging between 0 and 1

The effect of the pH on µ_max_ was expressed using the Cardinal Temperature and pH Model (CTPM) proposed by [30] according to Equation (3).

(3) $$CM_{n}\left(X\right)=\{\begin{array}{c} X\leq X_{min};0\\ \frac{{\left(X-X_{min}\right)}^{n}\left(X-X_{max}\right)}{{\left(X_{opt}-X_{min}\right)}^{n-1}\left\{\left(X_{opt}-X_{min}\right)\left(X-X_{opt}\right)-\left(X-X_{max}\right)\left[\left(n-1\right)X_{opt}+X_{min}-nX\right]\right\}}\\ X\geq X_{max};0 \end{array}$$

where *X* corresponds to environmental factors such as pH, temperature, and *n* values are 1 for pH and 2 for temperature, respectively.

Temperature and pH minimum cardinal values were determined by surface inoculation of MRS agar medium with drops of pre-cultivated strains on MRS broth at 37 °C for 8 h (achieving an absorbance of 1.4 at 600 nm). For the determination of the minimum pH value, MRS agar broth was buffered with phosphate buffer to achieve a pH ranging from 2.8 to 4, and incubated at 37 °C for 48 h in anaerobic conditions using an anaerobic jar and an Anaerocult kit (MERCK, F-67120, Molsheim, France) to produce CO_2_. For the minimum temperature, Petri dishes were incubated at 10 °C and 15 °C for two weeks. The temperature and maximum pH values were determined experimentally on batch cultures.

Optimum cardinal values for pH, temperature and µ_opt_ in MRS broth were adjusted from a set of experiments: with pH ranging from 4.0 to 9.5 at 37 °C and with temperature ranging from 20 °C to 55 °C, at pH 6.5.

The effect of the raw material (sorghum malt and flour dispersion) on µ_max_ was calculated as the ratio of the maximum growth rate in the raw material to the maximum specific growth rate in MRS broth at the same pH (6.5) and temperature (37 °C) as shown in Equation (4).

(4) $$\mu_{\max}ratio=\frac{\mu_{\max}{\left(6.5,\;37\;{}^{\circ}C\right)}_{\mathrm{RM}}}{\mu_{\max}{\left(6.5,\;37\;{}^{\circ}C\right)}_{\mathrm{MRS}}}$$

The effect of undissociated lactic acid on bacterial growth was determined using the Equation (5) proposed by [31]:

(5) $$\gamma \left(\left[AH\right]\right)=1-{\left(\frac{\left[AH\right]}{MIC}\right)}^{\alpha }$$

where [*AH*] represents the undissociated lactic acid concentration in mM, *MIC* is the minimum inhibitory concentration of the undissociated lactic acid and α reflects the shape of curves. We applied a value of 1 for α according to [31,32] for *Lactobacillus* strains.

The lag time (*λ*) is linked to the adaptation of a strain to a new environment. It is often considered as inversely correlated with μ_max_ [33], and the work to be done by the strain to adapt to a new environment is given by the product μ_max_·*λ*. Several expressions have been proposed to model this work and we used Equation (6) cited in [34]:

(6) $$\mu_{\max}.\lambda =\alpha +\beta \mu_{\max}$$

#### 2.5.2. Modeling Lactic Acid Production

The lactic acid content (g·L^−1^) in the model represents total lactic acid production without distinguishing between the D and L isomers, or between dissociated and undissociated lactic acid. The kinetics of lactic acid production was set directly proportional to bacterial growth as shown in Equation (7), as a simplification of Luedeking and Piret equation [29]:

(7) $$\frac{d(lactic)}{dt}=Y_{(\mathrm{lactic})/N}\times \mu N$$

where *Y*_(lactic)/N_ is the yield for lactic acid production over population (without distinction between D and L lactic acid), *N*, the population and µ is the growth rate.

#### 2.5.3. Modeling the pH Value of Gowé

A semi-mechanist model (Equation (8)) was used to predict the pH of sorghum flour depending on the lactic acid content of the medium ([*LA*], in g·L^−1^):

(8) $$\mathrm{pH}={\mathrm{pH}}_{acid}+\frac{{\mathrm{pH}}_{RM}-{\mathrm{pH}}_{acid}}{[LA]\times BP+1}$$

with pH*_acid_*, the pH of the lactic acid in pure water which was calculated from the classical dissociation equation of weak acids as shown in Equation (9), with lactic acid pKa of 3.8

(9) $${\mathrm{pH}}_{acid}=\frac{1}{2}\times \mathrm{pKa}-\frac{1}{2}\times \log\left(\frac{\left[LA\right]}{90}\right)$$

with pH*_RM_*, the pH of the raw material in pure water which was modeled with a power law equation (Equation (10)) from the sorghum flour concentration, [*RM*] (g·mL^−1^):

(10) $${\mathrm{pH}}_{RM}=\;\varphi \times {\left[RM\right]}^{-p}$$

To determine the coefficients φ and *p* of Equation (10), sorghum flour was dispersed in pure water at concentrations ranging from 0.04 to 0.4 g·mL^−1^ and the pH was measured. The coefficients were calculated by performing a linear regression after log-log transformation.

The buffering power (*BP*) of the medium was directly proportional to the sorghum flour content (g·mL^−1^) according to Equation (11):

(11) BP= ε× [RM]

To determine the coefficient *ε* of Equation (11), sorghum flour was dispersed in pure water at concentrations ranging from 0.04 to 0.4 g·mL^−1^. The dispersion was then titrated with 0.1 N lactic acid. The *ε* coefficient was determined by fitting the pH calculated with Equation (8) with the experimental pH for each sorghum flour content using non-linear regression.

### 2.6. Statistical Methods

The confidence intervals of means (at 95% probability) for non-linear and linear regressions were calculated using XLstat (Addinsoft, Paris, France). The cardinal values of Equation (3) were fitted using Tablecurve3Dsoftware (Jandel scientific, San Rafael, CA, USA); the confidence intervals were calculated using the Levenberg–Marquardt method.

## 3. Results and Discussion

### 3.1. Modeling Bacterial Growth

**Figure 2:**
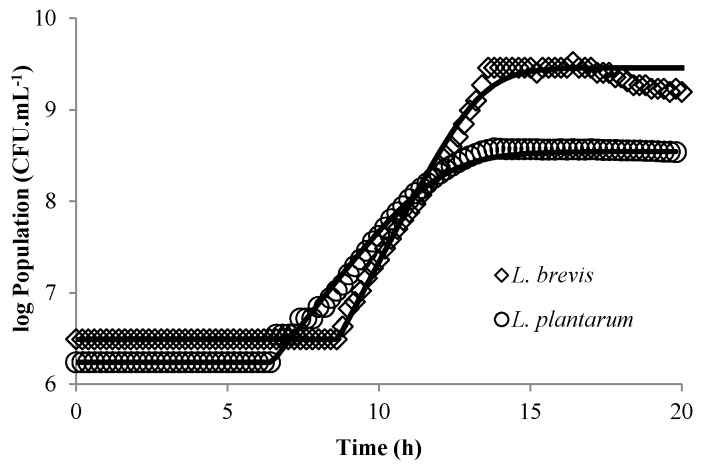
Growth of *Lactobacillus brevis* (◊) and *Lactobacillus plantarum* (○) in De Man Rogosa and Sharpe (MRS) broth at 37 °C, pH 6.5. Experimental data (symbols) and predicted data (continuous lines).

The primary logistic model in Equation (1) fits the experimental data well for both strains, as can be seen in Figure 2. Determining the growth rate with great accuracy for each experimental condition is essential for secondary modeling but the residual standard deviations of μ_max_ were 0.22 h^−1^ for *L. brevis* (five duplicates in similar conditions) and 0.28 h^−1^ for *L. plantarum* (four triplicates in similar conditions), respectively. This may be linked to perturbations of the signal in the case of high bacterial populations (as evidenced in Figure 2), due to cell aggregation and/or CO_2_ degassing, in particular for *L. brevis*.

In addition, large standard deviations for λ were observed; 1.6 h and 1.2 h for *L. brevis* and *L. plantarum*, respectively, for the same set of replications. Poor estimates of *λ* are indeed described in the literature, which can have several explanations [33]. In particular, a low level of inoculation, which was especially true for *L. brevis*, corresponded to the limit of detection of the sensor. Indeed [35] underlined the difficulty in determining *λ* at low levels of inoculation. Nevertheless, overall trends of the fermentation can be described satisfactorily even when the estimation of the *λ* is poor [36].

**Figure 3:**
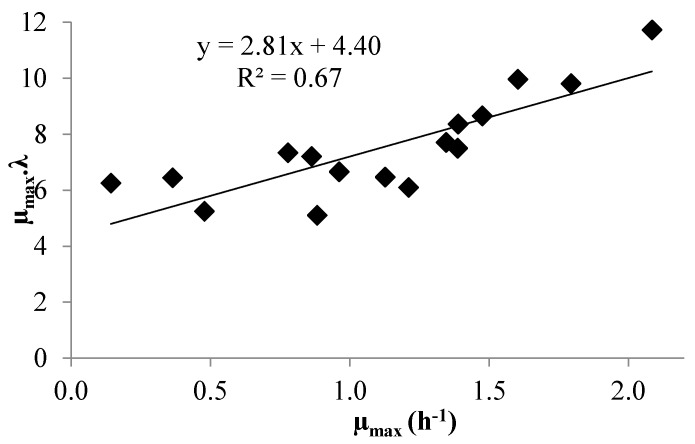
µ_ma**x**_·*λ* as a function of µ_max_ for *L. brevis.*

In the whole set of experiments, there was a weak linear correlation (*R*^2^ = 0.67 for both strains) between the work for adaptation to the environment (*λ*·μ_max_) and μ_max_, as shown in Figure 3 for *L. brevis*. The values of the regression parameters of Equation (6) were thus tainted by quite large uncertainties (Table 1). In contrast, [34] found a strong linear correlation between *λ*·*μ*_max_ and μ_max_(*R*^2^ = 0.99). This discrepancy was largely linked to our poor estimates of experimental *λ*.

The kinetic parameters of the two strains are listed in Table 1. The minimum and maximum cardinal values for pH and temperature were determined experimentally, and three parameters were adjusted: µ_opt_, pH_opt_ and T_opt_.

**Figure 4:**
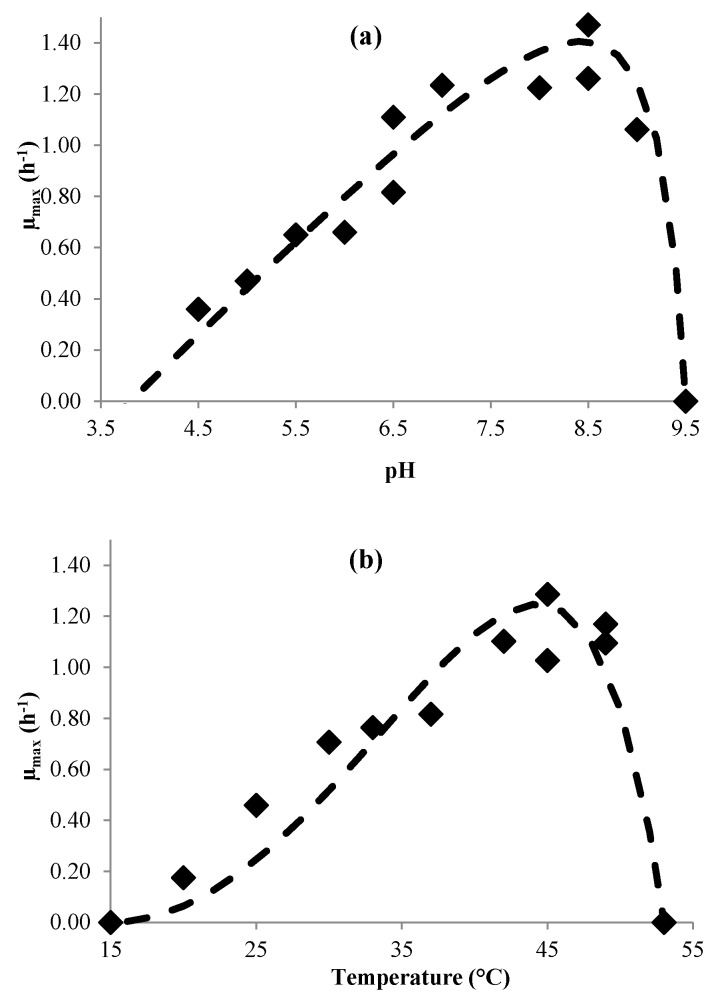
Effect of environment factors on growth rate of *L. brevis*. (**a**) pH effect and (**b**) temperature Experimental data (symbols) and predicted (continuous lines).

Although the confidence intervals for these parameters were quite large, the residual error of the predicted µ_max_ was moderate and a quite good fit of the predicted values. This can be observed in Figure 4.

The μ_opt_ values calculated with Equation (4) were 1.8 ± 0.2 h^−1^ and 1.4 ± 0.1 h^−1^ for *L. brevis* and *L. plantarum*, respectively. The μ_opt_ values determined for the two strains in MRS broth are slightly higher than those cited in literature for *L. plantarum* (0.64 h^−1^ by [37], and between 1.15 and 1.2 h^−1^ by [38]) and for *L. brevis* (between 0.9 and 1.4 h^−1^ by [39] and 0.68 h^−1^ by [40]).

Cardinal pH_min_ and pH_max_ values for *L. plantarum* are similar to those cited by [41,42,43,44]. However, the optimum pH values in the literature are generally lower than those found in our work. In [41] an optimum pH of 6 for *L. plantarum* was cited, while [39] cited an optimum pH of 5.5 for *L. brevis*. The optimum temperatures determined in this work are slightly higher than those cited in the literature. In [45,46], optimum temperatures were reported to be between 28 °C and 37 °C for *L. plantarum*, and [47] reported optimum temperatures between 30 °C and 37 °C for *L. brevis*.

Our strains thus evidenced quite original cardinal and optimum values compared with those cited in the literature. Lactobacillus strains can indeed present a wide range of genomic and technological properties, as shown for example for *L. plantarum* in red wine [48].

**Figure 5:**
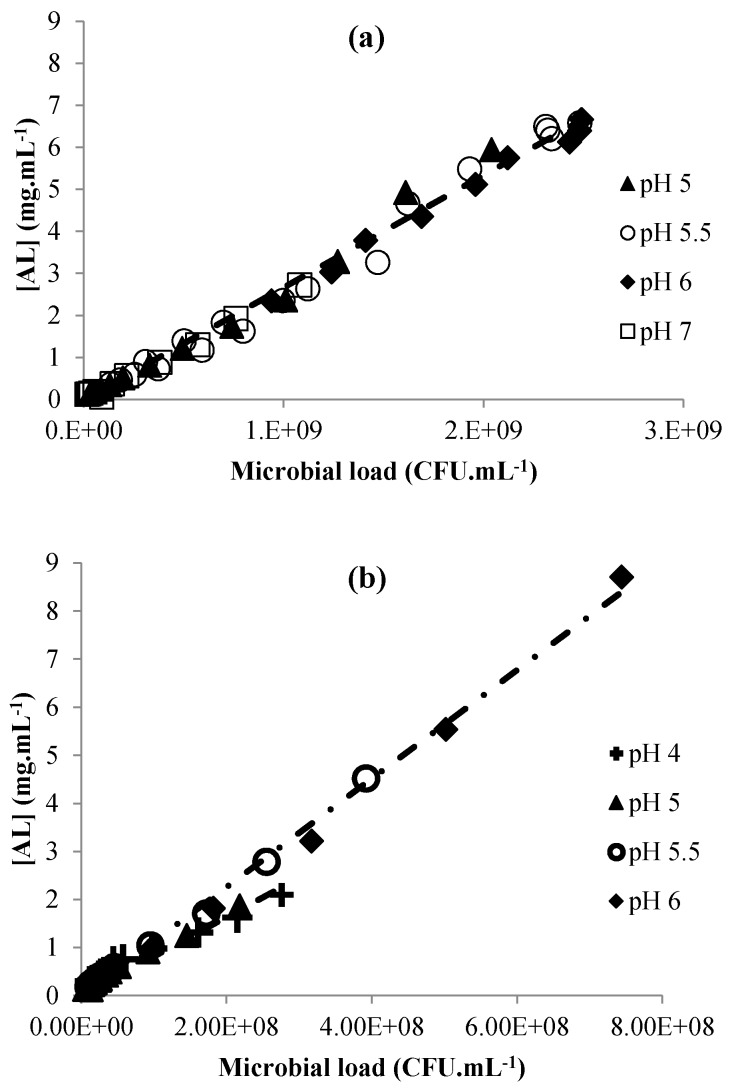
Regression between lactic acid concentration and microbial load for *L. brevis* (**a**) and *L. plantarum* (**b**) at pH 4 (**+**), 5 (▲), 5.5 (**○**), 6 (◆).

The minimum inhibitory concentration (MIC) of undissociated lactic acid was o 35.6 mM and 23.7 mM for *L. plantarum* and *L. brevis*, respectively. In the literature, the MIC of lactic acid are in the same range: [32] reported an MIC value of lactic acid of 53 mM for *L. helveticus*. It should be noted that the MIC of undissociated lactic acid did not perturb the determination of the *γ*([pH]). In our conditions, the maximum concentration of lactic acid at or above pH 5 in MRS inoculated with *L. brevis* or *L. plantarum* was of 6–8 mg/mL (Figure 5) which gave a *γ*([AH]) of 0.9 for both strains. It was of 0.8 for *L. plantarum* at pH 4, for which the maximum lactic acid concentration was 2 mg/mL. Indeed, these values, which are close to 1, did not really affect the growth rate and hence did not significantly bias the calculation of *γ*([pH]) which was, by comparison, 0.41 and 0.31 at pH 5.0 for *L. plantarum* and *L. brevis*, respectively.

### 3.2. Acid Lactic Production

The integration of Equation (7) means that the production of lactic acid is directly proportional to the size of the bacterial population. For *L. brevis* (Figure 5a) a constant production ratio of 2.71 (±0.08) × 10^−9^ mg·CFU^−1^ was found whatever the pH, between 5 and 7. The yield of glucose conversion to lactic acid (M/M) was close to 0.8 M/M at any pH, like the value cited by [49] for obligatory heterofermentative *Lactobacillus*. For *L. plantarum* (Figure 5b), we identified two distinct coefficients depending on the pH. When the pH was over 5, the ratio was 11.3 (±0.3) × 10^−9^ mg·CFU^−1^, but when the pH was under 5, the ratio was 8.5 (±0.6) × 10^−9^ mg·CFU^−1^. The yield of glucose conversion to lactic acid (M/M) was between 2 and 1.5 for *L. plantarum* at pH < 5 and pH > 5, respectively. These results are consistent with homofermentative metabolism [50]. The same effects of pH on lactic acid production and glucose conversion to lactate for *L. plantarum* were described by [51].

The characteristics of each strain are consistent with the common properties of their species. *L. plantarum*, a homolactic fermentative strain, is mesophilic (T_opt_ = 37.1 °C), acid tolerant (pH_min_ = 3.2) with a minimum inhibitory concentration (MIC) of undissociated lactic acid of 35.6 mM and a yield of glucose conversion to lactic acid (M/M) close to 2. *L. brevis*, a heterolactic fermentative strain, is mesophilic (T_opt_ = 44.6 °C), less acid tolerant (pH_min_ = 3.8) with a MIC of undissociated lactic acid of 23.7 mM and a yield of glucose conversion to lactic acid (M/M) close to 0.8.

### 3.3. Modeling the pH of Raw Material

**Figure 6:**
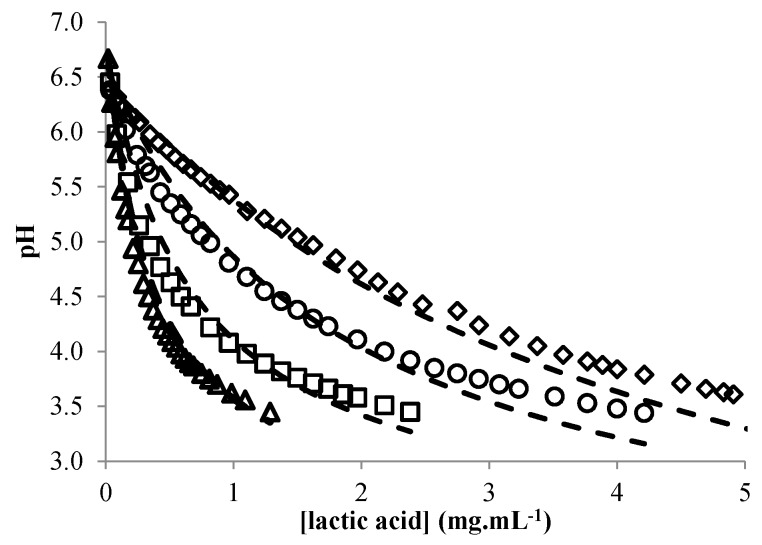
Changes in the experimental (symbols) and predicted (continuous lines) pH of sorghum flour dispersions (◇, 0.4 g/ML; **○**, 0.2 g/mL; □, 0.08 g/mL; △, 0.04 g/mL) with different lactic acid concentrations.

The pH of a dispersion of sorghum flour with various amounts of added lactic acid was modeled according to Equation (8) with a coefficient of determination of 0.98. The mean standard error of prediction was 0.17. The predicted pH was indeed very close to the actual pH (Figure 6) for values above 4, while for lower values, the predicted pH was slightly under-estimated.

**Figure 7:**
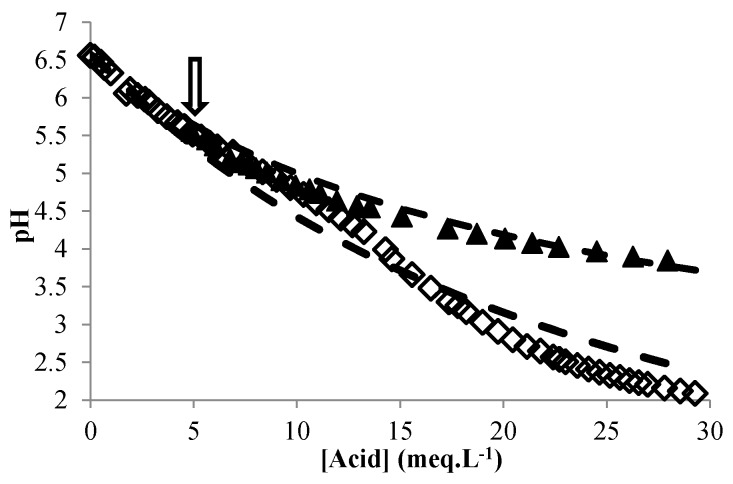
Changes in the experimental (symbols) and predicted (continuous lines) pH of sorghum flour dispersion with the addition of sulfuric acid (◇, 2 g/mL) and of malt dispersion with the addition of lactic acid (▲, 2 g/mL) (the arrow points to the initial pH of the malt dispersion).

It was also possible to apply the model when the dispersion of sorghum flour was titrated with sulfuric or acetic acid, leading to a change in Equation (9) by calculating pH for a strong acid or changing the pKa of the weak acid, and calculating the acidity in meq·L^−1^ and expressing ε in mL·meq^−1^. Without modification of the parameters, the model fit quite well (Figure 7) for strong or weak acid. In addition, it was also possible to use it for a dispersion of sorghum malt by simply taking the initial lactic acid content of the malt into account. This clearly showed that pH_RM_ and BP are quite robust parameters that do not change significantly after malting and that can thus be used for different sorghum flours. Quite similar semi-empirical approaches for predicting the pH of fermenting media have been proposed by [20,34]. The advantage of our model is that provided the initial lactic acid content is known, the pH can be calculated irrespective of the concentration of raw material and with various types of materials (sorghum flours or malts). A model with more extensive applications (wider range of raw materials) could be developed in the future using the approach of [52].

### 3.4. Validation of the Global Model

The implementation of the models and the corresponding Equations from (1) to (9), led to a global model that was tested in real conditions during the manufacture of gowé at laboratory scale in conditions close to those of the traditional process. To avoid interactions with the wild microbial flora such as lactic bacteria or yeasts, the raw materials were decontaminated by irradiation. During fermentation, the population of lactic acid bacteria was enumerated by plate counting on MRS agar medium because the turbidity of the suspension of flour and malt indeed prevented the use of an Optek turbidity sensor.

In the first step, we determined the µ_max_ ratio with the raw material. These values are higher than those calculated by the gamma model in MRS broth in the same conditions. This gave a µ_max_ ratio of over 1 for both strains (Table 1). This ratio was thus used as a multiplier to calculate of changes in the population of lactobacilli in the raw material from the model developed in MRS. The free sugar (glucose and maltose) content of the raw material indeed ranged from 20 to 30 g·L^−1^ throughout the process of fermentation of the raw material, greater than 16 g·L^−1^ for MRS broth at the beginning of the fermentation and then declined to 0 at the end.

**Figure 8:**
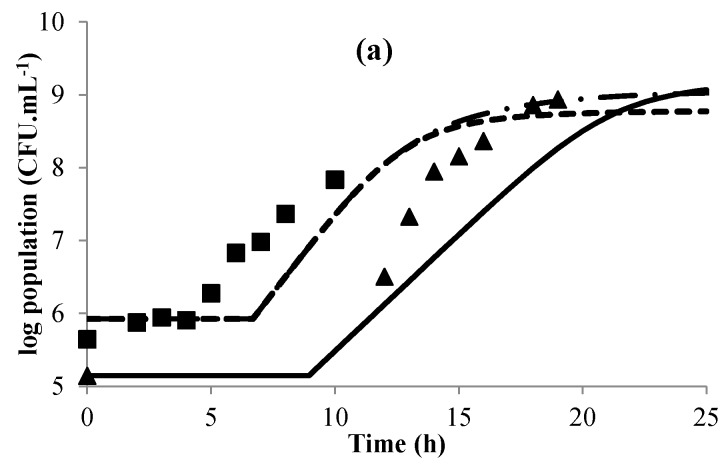

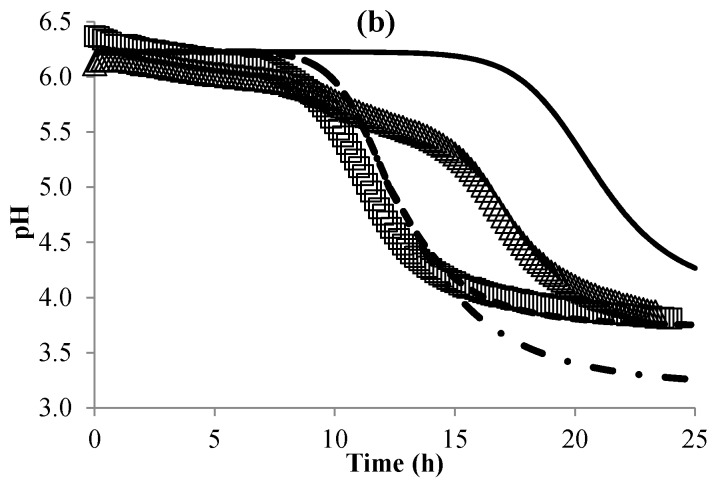
Changes in the log population (**a**) and in pH (**b**) for the validation tests. Experimental data for *L. plantarum* (■, □) and *L. brevis* (▲, △). Modeled curves for *L. plantarum* (complete model, ---; model without dissociated lactic acid gamma parameter -·-) and *L. brevis* (complete model, - - -; model without dissociated lactic acid gamma parameter, -).

In the second step, we performed the fermentation at 30 °C (close to ambient temperature in Benin), with no control of pH but under gentle agitation. Figure 8 compares the experimental data obtained for the fermentation of the raw material inoculated with each strain with the data predicted by the global model we developed. For the bacterial growth of *L. plantarum* (Figure 8a), the model predicts a lag phase of about 7 h, longer than the experimental lag phase, which was between 4 and 5 h. Nevertheless, it is possible to consider that the models fit the experimental data quite well because the difference between the experimental data and the calculated values is less than 1 log unit, which seems acceptable considering the method of enumeration. An improvement of the model is however necessary to better predict the lag phase, which is indeed tainted by large uncertainties (see above). Figure 8b shows changes in pH during the fermentations of each strain. The model indeed predicted very different behavior for each strain: *L. plantarum*, a homolactic strain, results in much more rapid acidification than *L. brevis*, a heterolactic strain. For *L. plantarum*, the pH predicted by the model fits the experimental pH very well, with only a later start of acidification, due to the prediction of a longer lag phase. It should be noted that when γ([AH]) was omitted in the model, the predicted pH largely deviated from the experimental pH after 15 h of fermentation. This clearly shows that the concentration of undissociated lactic acid stops bacterial growth, not pH alone. Similarly [32,53,54] demonstrated that the undissociated lactic acid is the main inhibitor of the growth of lactobacilli. However, this effect was much less clear on bacterial growth (Figure 8a) as it is expressed in log values. In the case of *L. brevis*, the model does not fit the experimental data well.

In the experimental data, the pH dropped within two steps. The first drop of pH occurred between 6 and 15 h; it cannot be linked to lactic acid production for maintenance of *L. brevis*, as its level of lactic acid production for maintenance would have to be 10 times higher than that needed for growth in order to explain the observed drop in pH, whereas it is generally 10 times lower [34] and consequently neglected by most authors. This drop may be linked to the growth of yeasts whose residual population was of 1.3 × 10^4^ CFU·g^−1^ in malted and non-malted irradiated flours i.e., 6.10^3^ CFU·mL^−1^ of gowé. The second drop, linked to the growth of *L. brevis*, was predicted with a delay by the model. This may be linked to overestimation of lag time by the model, like for *L. plantarum*. The difficulty of the model to correctly predict the latent phase is an obstacle that needs to be overcome to improve the quality of the prediction. This will be addressed in particular by improving the accuracy of determination of lag time (increase in the level of inoculation). Nevertheless, the model can already be used to test inoculation (choice of the inoculation strain, level of inoculation) and fermentation conditions (temperature and duration of fermentation) that directly affect the amount of lactic acid and hence the drop in pH, which will directly affect the quality of final gowé. A rapid acidification, after inoculation with homolactic *L. plantarum* will lead to a safer product, but as the acidification is detrimental to starch amylolysis [28], this will lead to a less sugary product. A global model, integrating models for amylolysis and yeast growth, is thus necessary to define the optimal fermentation conditions for producing safe and appreciated gowé.

## 4. Conclusions

A global model of the lactic fermentation of gowé was developed on a synthetic medium step by step (block by block) through the implementation of different specific models (blocks) that can predict (i) the kinetics of bacterial growth in different temperature and pH conditions; (ii) the production of lactic acid; (iii) the pH of the medium depending on the concentration of lactic acid; and (iv) the inhibition of the growth of lactic strains due to the concentration of undissociated lactic acid. The global model was used to simulate the behavior of two commercial strains during the fermentation of sorghum flour and malt to produce gowé. Greater attention will be paid in the future to the determination of the kinetic parameters of the two strains, in particular to improve the determination of the lag phase, which should make it possible to improve the performance of the model as a whole.

The global model can already predict the acidity of gowé, but to take into account the sweet taste, which is also a major quality attribute of gowé, the global model can be enriched by adding blocks to model yeast growth and sugar release by amylases whose activity depends on temperature, pH and sugar content.

## Figures and Tables

**Table 1:** Parameters of the models of µmax, lactic acid production rate and pH. CI = confidence intervals (α = 0.05).

Modeled Variable	Equation	Parameter	Value ± CI (*L. brevis*)	Value ± CI (*L. plantarum*)
µ_max_	(3)	pH_min_ pH_max_ pH_opt_ µ_opt_ (h^−1^) T_min_ (°C) T_max_ (°C) T_opt_ (°C)	3.8 9.3 8.3 (±0.3) 1.8 (±0.2) 15 53 44.6 (±1.2)	3.2 9.2 8.3 (±0.3) 1.4 (±0.1) 12 52 37.1 (±3.2)
	(4)	µ_max_ ratio	1.57 (±0.1)	1.36 (±0.06)
MIC	(5)	[*AH*] mM	23.73	35.59
λ	(6)	α	4.4 (±1.2)	1.17 (±1.4)
		β	2.8 (±1.0)	5.6 (±1.5)
Lactic acid production rate	(7)	Y_(lactic)/N_ (10^−9^ mg·CFU^−1^)	2.71 (±0.08)	11.3 (±0.3) * 8.5 (±0.6) ^$^
pH_RM_	(10)	φ	6.30 (±0.006)	
		ε	0.028 (±0003)	
BP	(11)	(mL·g^−1^)	0.16 (±0.02)	

* for pH > 5; ^$^ for pH ≤ 5.
